# Metabolic reprogramming-mediated therapeutic resistance in renal cell carcinoma: Mechanisms and targeted intervention strategies

**DOI:** 10.1016/j.isci.2026.117279

**Published:** 2026-09-01

**Authors:** Han Wu, Xinxin Gan, Siyi Wang, Ruoyao Wang, Jiatao Hu, Linhui Wang, Jiazi Shi

**Affiliations:** 1 Department of Urology, The Second Affiliated Hospital of Naval Medical University, Shanghai, China; 2 Department of Urology, The First Affiliated Hospital of Naval Medical University, Shanghai, China

**Keywords:** renal cell carcinoma, metabolic reprogramming, therapy, drug resistance, minimally invasive surgery

## Abstract

Renal cell carcinoma (RCC) is one of the most frequent urological tumors and exhibits metabolic reprogramming alongside genetic mutations. RCC cells reprogram pathways involved in glucose, lipid, and amino acid metabolism to meet their energy and anabolic demands. In recent years, targeted drugs, immunotherapy, and radiotherapy have significantly improved the prognosis of patients with metastatic RCC. Nevertheless, the development of resistance remains a major cause of treatment failure and disease progression in metastatic RCC. A deeper understanding of the metabolic mechanisms underlying therapy resistance is critical for refining therapeutic strategies. The molecular mechanism of metabolic reprogramming involved in therapy resistance is comprehensively discussed. Novel strategies for overcoming therapy resistance of RCC are proposed, including the development of new drugs targeting metabolism, nanoparticles co-loaded with anti-tumor drugs, the application of combination therapies, and personalized medicine approaches. In conclusion, this review provides a thorough overview of metabolic alterations in RCC and their role in therapy resistance, highlighting the potential for developing new treatments for RCC.

## Graphical abstract

**Figure:**
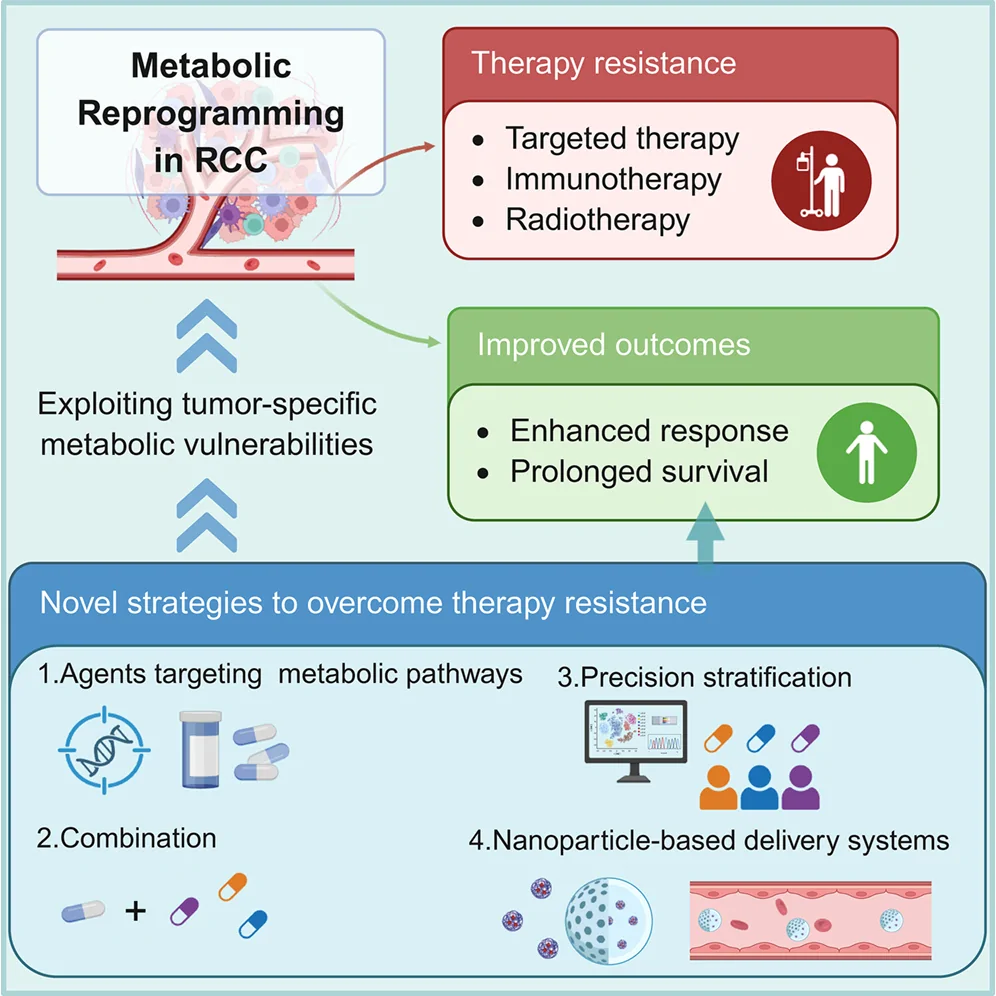

## Teaser

Biological sciences; Cell biology

## Introduction

Metabolic reprogramming is a hallmark of cancer, defined as the alteration of metabolism in cancer cells. Unlike normal cells, cancer cells proliferate rapidly, requiring abundant energy and biomolecules, enhanced stress tolerance, and adaptations to the tumor microenvironment. To meet these demands, cancer cells remodel metabolic pathways.^1^^,^^2^ Renal cell carcinoma (RCC) is one of the most frequent urological tumors and is often regarded as a metabolic disease.^3^^,^^4^ Commonly mutated genes and altered signaling pathways in RCC are involved in regulating various metabolic processes, including glycolysis, the TCA cycle, fatty acid oxidation, and amino acid metabolism.^3^^,^^5^ For RCC in the early stage, tumor resection can be curative with a good prognosis. However, advanced or metastatic RCC remains associated with a poor prognosis. Multiple treatment modalities, including tyrosine kinase inhibitors (TKIs), immunotherapy, and radiotherapy, have greatly improved the overall survival in advanced RCCs. Unfortunately, most patients eventually develop therapy resistance.^6^^,^^7^^,^^8^ Accumulating evidence in recent years has revealed a close relationship between cancer metabolic reprogramming and therapy resistance, as cancer cells alter metabolic pathways to diminish drug effects and promote resistance.^9^^,^^10^^,^^11^^,^^12^

In the present review, we systematically reviewed key events in RCC metabolic reprogramming and their contribution to therapy resistance. Furthermore, novel drugs targeting metabolic reprogramming are also covered. We propose possible therapeutic approaches for overcoming therapy resistance. We hope that recent advances and insights will provide inspiration for treating therapy-resistant RCC.

## Reprogrammed metabolites and related pathways in RCC

### Carbohydrate metabolism

Glucose, the core substrate for systemic energy supply and biosynthesis, maintains cellular metabolic and redox homeostasis through glycolysis and the tricarboxylic acid (TCA) cycle, with the pentose phosphate pathway (PPP) serving as a complementary anabolic bypass. The kidney regulates whole-body glucose balance via tubular glucose reabsorption, urinary glucose excretion, and renal gluconeogenesis, and abundantly expresses GLUTs and SGLTs to meet its intrinsic high glycolytic demand.

RCC cells manifest the prototypical Warburg effect, whereby anaerobic glycolysis is prioritized even under normoxic conditions, unlike normal cells that depend on standard glycolysis linked to mitochondrial oxidative phosphorylation (OXPHOS).^4^^,^^13^ Distinct from standard glycolysis that delivers pyruvate to the TCA cycle for complete oxidation, anaerobic glycolysis diverts nearly all cytoplasmic pyruvate away from mitochondria; instead, pyruvate is rapidly reduced to lactate via lactate dehydrogenase A (LDHA), ultimately leading to massive lactate accumulation. Excessive lactate produced by anaerobic glycolysis via the Warburg effect serves as a central driver in shaping the immunosuppressive tumor microenvironment (TME) of RCC.^14^^,^^15^ On one hand, lactate secretion induces extracellular acidification of the TME; on the other hand, sustained anaerobic glycolysis leads to aberrantly elevated glucose uptake by tumor cells, substantially depleting local glucose reserves and impairing the metabolic fitness of tumor-infiltrating T lymphocytes, thereby suppressing their antitumor effector functions.^16^^,^^17^ Additionally, RCC cells upregulate indoleamine 2,3-dioxygenase 1 (IDO1), which depletes tryptophan—an essential amino acid for T cell activation—and generates kynurenine. Kynurenine further inhibits cytotoxic T cell activity and promotes the proliferation of immunosuppressive regulatory T cells (Tregs).^18^ Collectively, lactate-induced acidification, glucose nutrient depletion, and accumulation of immunosuppressive metabolites establish a metabolically hostile TME that blunts anti-tumor immune responses. Concomitant with the downregulation of the TCA cycle and OXPHOS, the PPP is typically upregulated in RCC.^19^^,^^20^^,^^21^ Hyperactivated PPP flux generates NADPH to scavenge reactive oxygen species and preserve intracellular redox homeostasis, while concurrently supplying ribose-5-phosphate for *de novo* nucleotide biosynthesis to sustain rapid tumor proliferation.^22^^,^^23^

**Figure 1:**
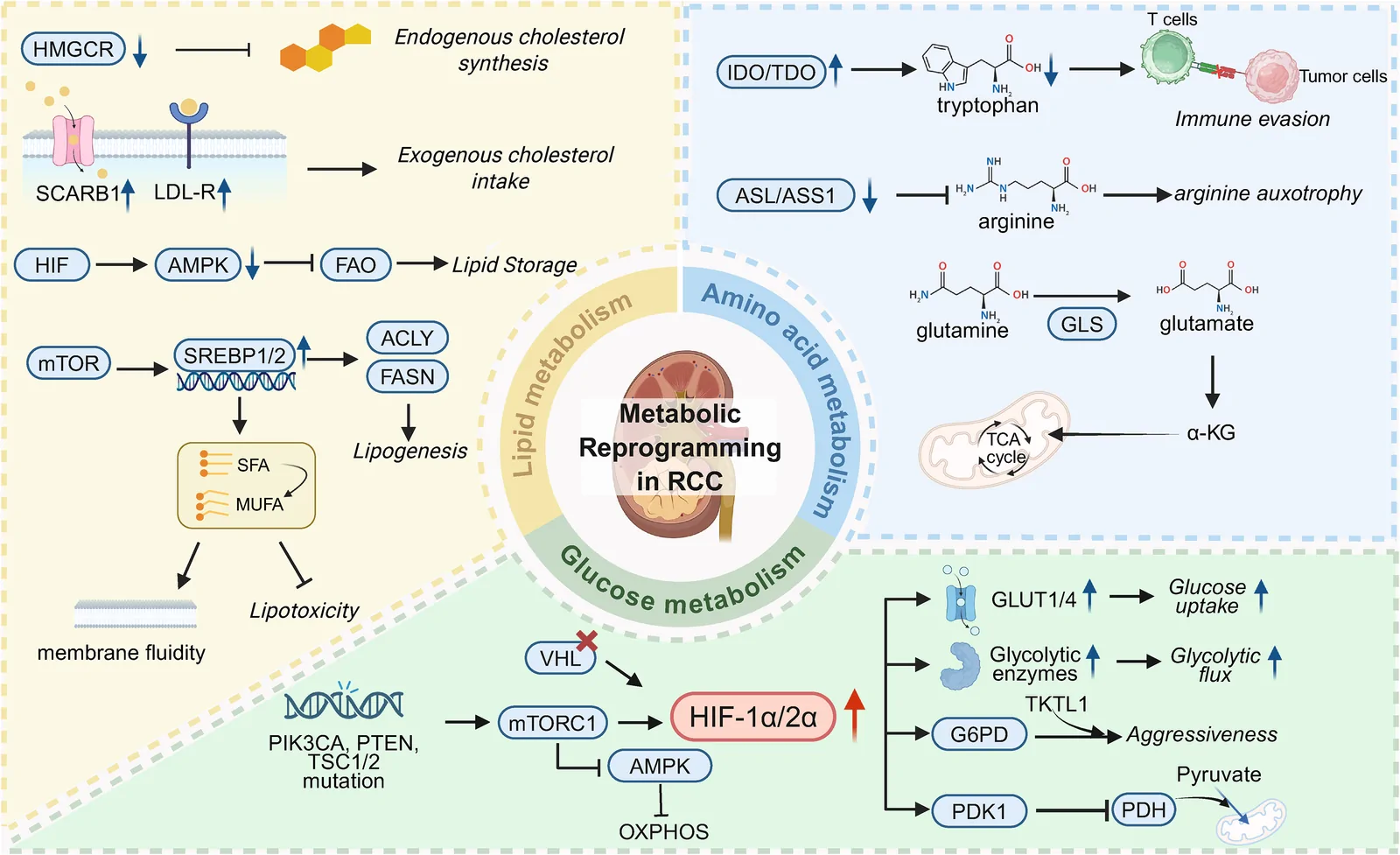
Metabolism reprogramming regulation of genes and related signaling pathways in RCC HMGCR, HMG-CoA reductase; LDL-R, low-density lipoprotein receptor; HIF, hypoxia inducible factor; AMPK, AMP-activated protein kinase; FAO, fatty acid β-oxidation; SREBP1/2, sterol regulatory element-binding protein; ACLY, ATP citrate lyase; FASN, fatty acid synthase; SFA, saturated fatty acids; MUFA, monounsaturated fatty acids; IDO, indoleamine 2,3-dioxygenase; TDO, tryptophan-2, 3-dioxygenase; GLS, glutaminase; ASS1, argininosuccinate synthase-1; ASL, argininosuccinate lyase; α-KG, alpha-ketoglutarate; TSC1, 2, tuberous sclerosis complex1, 2; PTEN, phosphatase and tensin homolog; VHL, von Hippel Lindau; OXPHOS, oxidative phosphorylation; GLUT, glucose transporter; G6PD, glucose-6-phosphate dehydrogenase; PDH, pyruvate dehydrogenase; PDK1, pyruvate dehydrogenase kinase-1.

The metabolic reprogramming of glucose in RCC is intimately linked to the Warburg effect and a state of “pseudohypoxia”, wherein multiple signaling pathways play pivotal roles (Figure 1).^3^^,^^5^ The most frequent initiating event is the inactivation of the von Hippel-Lindau (VHL) tumor suppressor gene. This loss leads to the stabilization of hypoxia-inducible factors (HIF-1α and HIF-2α) even under normal oxygen conditions, a state known as pseudohypoxia. The stabilized HIFs then upregulate glucose transporters (GLUT1/4) and glycolytic enzymes (HK2, LDHA, PGK1), shunting glucose metabolism toward lactate production and thereby fostering the immunosuppressive TME. Beyond the canonical VHL/HIF axis, mutations in PIK3CA, PTEN, or TSC1/2 trigger excessive mTOR hyperactivation, which enhances HIF-1α translation. This regulatory cascade consequently upregulates GLUT1 expression and glycolytic flux while inhibiting OXPHOS. Furthermore, amplification of the c-MYC oncogene often cooperates with HIF-2α to upregulate genes involved in glutaminolysis and nucleotide synthesis, thereby reinforcing the glycolytic dependency of RCC cells.^3^^,^^5^^,^^24^^,^^25^ These oncogenic inputs, compounded by marked depletion of fructose-1,6-bisphosphatase (FBP1)—a critical negative regulator of glycolytic flux and the Warburg effect,^26^ converge to lock RCC cells into an irreversible glycolytic state. On the other hand, HIF-1α drives the PPP by inducing its rate-limiting enzyme G6PD alongside transketolase-like 1 (TKTL1). Studies have shown that aberrant G6PD overexpression is prevalent in RCC and associated with poor prognosis, and recent preclinical evidence demonstrates that G6PD inhibition exerts cell line-specific antitumor effects.^27^^,^^28^^,^^29^

**Figure 2:**
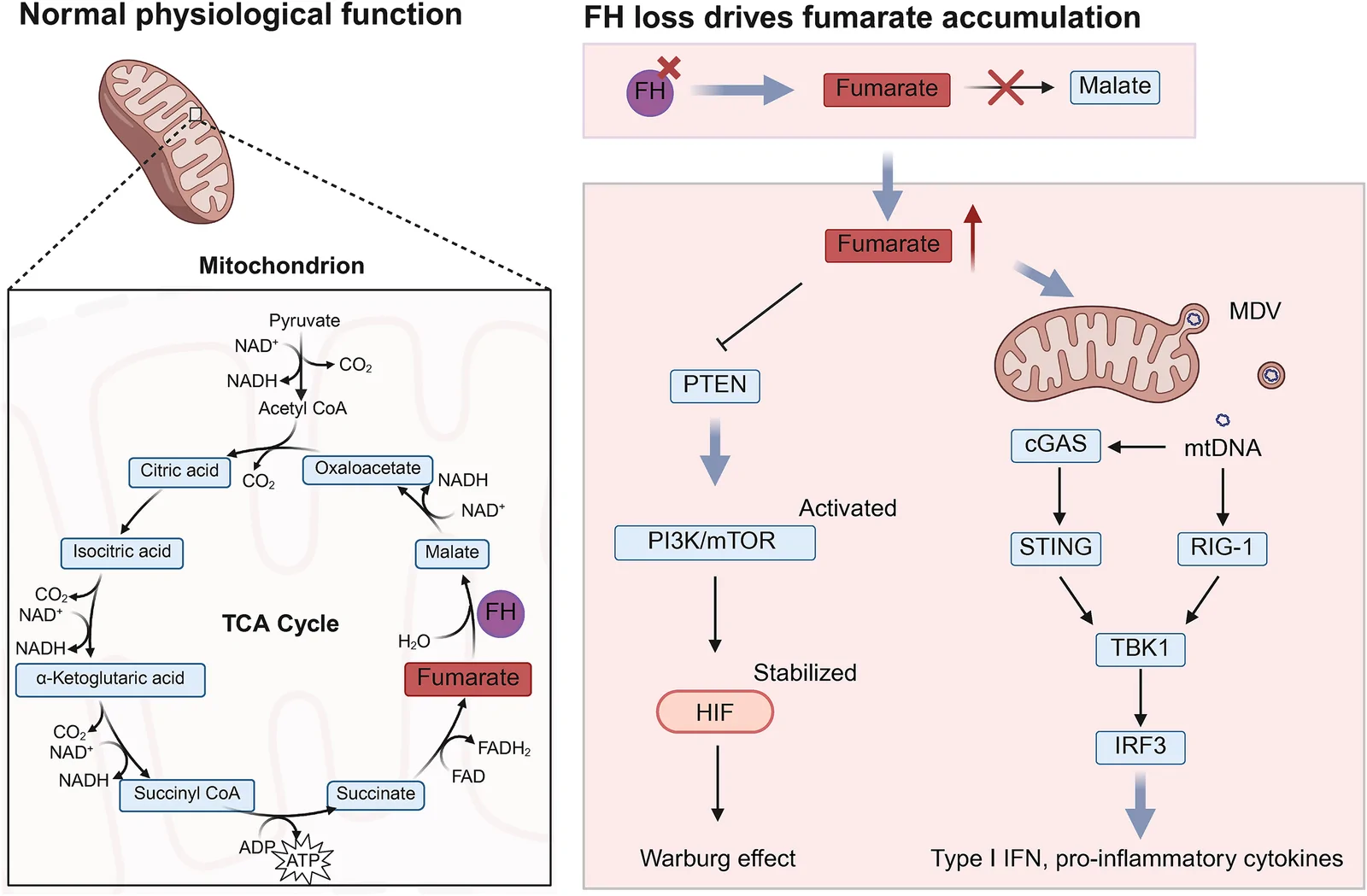
FH loss-mediated fumarate accumulation drives metabolic reprogramming FH, fumarate hydratase; MDV, mitochondrial-derived vesicle.

Concurrently, mitochondrial metabolic pathways are suppressed in RCC.^30^ Fumarate, an intermediate of the mitochondrial TCA cycle, participates in the oxidation of carbohydrates, amino acids, and fatty acids; it also serves as a byproduct of the urea cycle, as well as amino acid and nucleotide metabolism.^31^ In the TCA cycle, succinate is converted to fumarate catalyzed by succinate dehydrogenase (SDH), and the resultant fumarate is subsequently transformed into malate via fumarate hydratase (FH) (Figure 2). Notably, mutations in key TCA cycle enzymes such as FH and SDH define specific hereditary forms of RCC (FH-deficient and SDH-deficient RCC). In these cases, metabolite accumulation (e.g., fumarate in FH-deficient RCC) can directly inhibit tumor suppressors such as PTEN, further activating oncogenic pathways.^32^ Beyond oncogenic signaling activation driven by accumulated oncometabolites, loss of FH initiates a unique metabolic-immune crosstalk cascade that accounts for the origin of the characteristic chronic inflammatory TME specific to FH-deficient RCC. Massive intramitochondrial fumarate accumulation resulting from FH deficiency remodels mitochondrial architecture and triggers SNX9-dependent generation of mitochondrial-derived vesicles (MDVs), which mediate the efflux of mitochondrial DNA (mtDNA) into the cytosol. Cytosolic mtDNA subsequently activates dual innate immune sensors cGAS and RIG-I, converging on the TBK1–IRF3 axis to sustain persistent type I interferon and pro-inflammatory cytokine secretion, thereby establishing a chronic low-grade inflammatory milieu.^33^ This mitochondrial dysfunction is compounded by suppressed pyruvate dehydrogenase (PDH) activity, which is mediated by HIF-induced pyruvate dehydrogenase kinase 1 (PDK1), effectively blocking pyruvate’s entry into the mitochondria. Meanwhile, the impairment of OXPHOS is associated with the downregulation of complexes such as ATP synthase (complex V).^34^ As both the TCA cycle and OXPHOS occur in the mitochondria, the regulation of mitochondrial biogenesis is crucial. The master regulator of this process, PGC-1α, is suppressed in VHL-deficient RCC. Low PGC-1α expression is correlated with poor patient outcomes, and its repression by factors such as MYB-binding protein 1A (MYBBP1A) can inhibit tumor growth, highlighting the complex role of mitochondrial regulation in RCC progression.^35^^,^^36^

### Lipid metabolism

Lipid metabolic reprogramming acts as a core driver of RCC pathogenesis, affecting lipid uptake, storage, homeostasis, and membrane biogenesis to support rapid tumor growth. Etiologically, obesity is a well-established risk factor for RCC.^37^^,^^38^ Massive lipid deposition within lipid droplets (LDs), predominantly cholesterol and cholesterol esters (ChE), represents the most distinctive pathological signature of clear-cell RCC (ccRCC).^39^ This reflects a metabolic shift where energy production from lipids is reduced, and lipids are diverted toward storage.^40^

This metabolic shift is primarily manifested in the dysregulation of fatty acid turnover, which is divided into two reciprocal branches: catabolism and anabolism. Fatty acids can be oxidized to acetyl-CoA, which enters the TCA cycle.^41^ Conversely, acetyl-CoA can be catalyzed into fatty acids by fatty acid synthase (FASN). Fatty acids also serve as backbones for the synthesis of many biological macromolecules such as hormones, triglycerides, and phospholipids, which are necessary for cell growth.^42^ Normally, fatty acid β-oxidation (FAO) is impaired in RCC, while fatty acid synthesis is enhanced.^43^ In addition, glucose-derived citrate was converted to acetyl-CoA to sustain excessive lipid synthesis. RCC represses endogenous cholesterol synthesis enzymes (e.g., HMGCR) but upregulates LDL receptors (LDL-R) and scavenger receptor B1 (SCARB1) to import circulating cholesterol.^44^ Elevated levels of cholesterol esters, fatty acylcarnitines, and carnitine are also observed in patients with RCC or tissues. RCC accumulates ChE in lipid droplets, which provides building blocks for membranes and signaling molecules during proliferation.^45^ Beyond fatty acid and cholesterol pathways, glycerophospholipid-linked arachidonic acid metabolism is also remodeled. In ccRCC cells lacking VHL, c-MYC increases triglyceride content, promoting LD formation in a glutamine-dependent manner.^46^

This metabolic reprogramming is driven by key signaling pathways. The ATP-citrate lyase (ACLY) and FASN are overexpressed to facilitate lipogenesis.^47^ Critically, the VHL/HIF axis regulates cholesterol uptake, fatty acid synthesis, and lipid droplet formation. HIFs also suppress FAO by downregulating carnitine palmitoyltransferase 1A (CPT1A), thereby promoting lipid storage.^48^^,^^49^ Concurrently, the mTOR pathway enhances lipid biosynthesis by upregulating sterol regulatory element-binding proteins (SREBPs), which are master transcription factors for lipogenic enzymes such as ACLY, FASN, and stearoyl-coenzyme A desaturase-1 (SCD1).^50^ SCD1 generates monounsaturated fatty acids, a process that induces lipid desaturation and helps prevent ferroptosis.^51^ The overexpression of SREBP1 in RCC drives this desaturation, converting saturated to monounsaturated fatty acids to maintain membrane fluidity and prevent lipotoxicity.^52^^,^^53^

### Amino acid metabolism

Amino acid metabolism is crucial for RCC growth and survival.^54^ Like glucose and lipid pathways, amino acid metabolism is reprogrammed to support tumor proliferation, immune evasion, and therapy resistance. Key roles of amino acid metabolism in RCC include fueling biosynthesis (nucleotides, proteins), maintaining redox homeostasis, modulating immune response, and driving epigenetic and signaling changes.^3^^,^^55^ Several risk signatures based on amino acid metabolism genes have been proposed, stratifying patients with RCC into high- or low-risk groups and predicting therapy responses and prognosis.^56^^,^^57^

Glutamine is a major nutrient for protein and lipid synthesis. It is also the precursor of glutathione, an important antioxidant that protects cancer cells from oxidative stress.^58^ The enzyme glutaminase (GLS) catalyzes the conversion of glutamine to glutamate; STAT1 can transcriptionally activate GLS1.^59^ This glutamate can be utilized for cytoplasmic protein synthesis or further converted to α-ketoglutarate (α-KG) by glutamate dehydrogenase (GDH), thereby replenishing the tricarboxylic acid (TCA) cycle.^60^ RCC exhibit markedly enhanced glutamine consumption relative to normal kidney tissue.^61^ Proteomic and metabolomic studies detect enrichment of metabolites in glutamine and GSH/GSSG pathways in RCC.^27^^,^^55^ Increased uptake of the glutamine analog 18F-(2S,4R)-4-fluoroglutamine is observed in orthotopic mouse models of renal tumors using PET imaging.^21^ Mechanistically, RCC upregulates the glutamine importer ASCT2 (alanine-serine-cysteine transporter 2) and catalytic enzyme GLS1 to sustain malignant growth.^62^ Inhibiting ASCT2 (with V9302) or GLS (with CB-839), alone or combined with other drugs, shows anti-tumor effect in ccRCC.^63^ Furthermore, glutamine-derived aspartate serves as a carbon source for pyrimidine biosynthesis.

Tryptophan, an essential amino acid, is primarily metabolized through three major pathways: serotonin, indoleacetate, and kynurenine (KN). The KN pathway is the dominant route, consuming the majority of dietary tryptophan in RCC.^64^ Metabolites of this pathway exert potent immunosuppressive effects within the tumor microenvironment.^65^ Increased tryptophan consumption via the KN pathway is a key contributor to immune evasion in RCC. This is driven by the overexpression of key enzymes in the KN pathway, namely indoleamine 2,3-dioxygenase (IDO) and tryptophan 2,3-dioxygenase (TDO).^66^

Arginine, a semi-essential amino acid involved in multiple metabolic processes, is synthesized from citrulline via the urea cycle enzymes argininosuccinate synthase-1 (ASS1) and argininosuccinate lyase (ASL).^67^ In RCC cells, the expression of ASS1 and ASL is frequently epigenetically suppressed. This results in arginine auxotrophy, forcing the cancer cells to depend on an external supply of arginine. Consequently, therapeutic strategies employing arginine deprivation, such as using arginine deiminase (ADI), have shown promise in treating RCC.^61^

In addition, serine and glycine metabolism are deeply involved in RCC progression. These non-essential amino acids support one-carbon metabolism for nucleotide synthesis and methylation reactions, contributing to redox balance (via NADPH and GSH production).^64^ Key enzymes in serine/glycine biosynthesis and one-carbon metabolism, such as PHGDH, SHMT2, and MTHFD2, are therapeutic targets for eradicating RCC resistance.^68^ Dysregulated catabolism of branched-chain amino acids (BCAAs; leucine, isoleucine, valine) is another characteristic metabolic signature of RCC.^4^ Notably, suppressed expression of BCAA catabolism-related genes is strongly associated with worse survival in ccRCC, highlighting its prognostic value.^69^

## Metabolic reprogramming in therapy resistance of RCC

**Figure 3:**
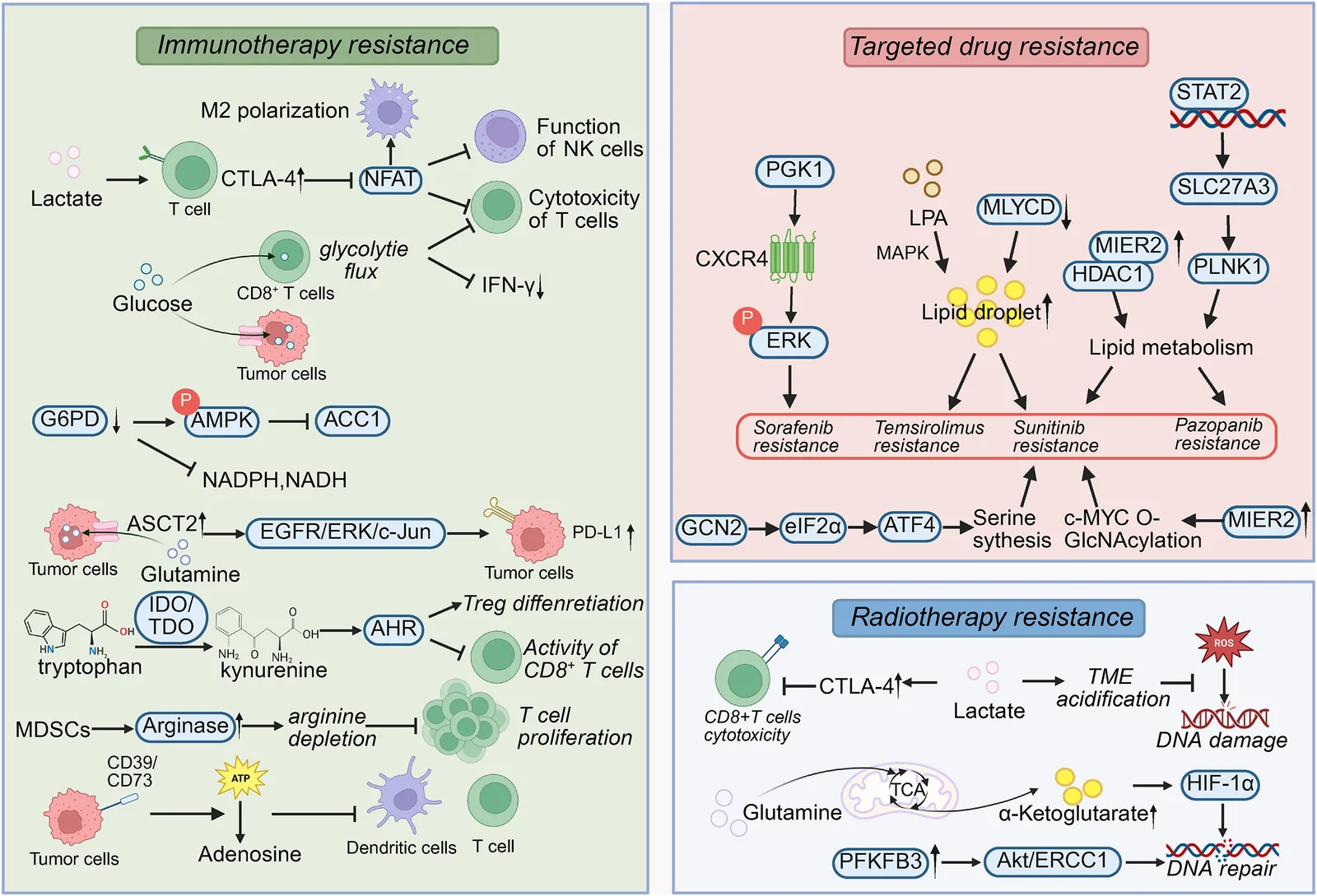
Metabolic reprogramming in therapy resistance of RCC G6PD, glucose-6-phosphate dehydrogenase; AMPK, AMP-activated protein kinase; IDO, indoleamine 2,3-dioxygenase; TDO, tryptophan-2, 3-dioxygenase; MDSCs, myeloid-derived suppressor cells; AHR, aryl hydrocarbon receptor; PGK1, phosphoglycerate kinase 1; MLYCD, malonyl-CoA decarboxylase; MIER2, mesoderm induction early response 2; HIF, hypoxia inducible factor; PFKFB3, 6-phosphofructo-2-kinase/fructose-2,6-biphosphatase 3.

Treatment of mRCC has undergone dramatic changes in recent years. Emerging therapies have significantly improved patient prognosis. Management of mRCC is highly personalized and systemic, requiring effective combination strategies.^70^ Conventional chemotherapy has shown limited efficacy for patients with RCC. Targeted therapy with TKIs (sunitinib, axitinib, pazopanib, cabozantinib, lenvatinib) and mTOR inhibitors (everolimus, temsirolimus) is widely used in first- and second-line treatments, blocking tumor angiogenesis and nutrient supply. Immunotherapy for RCC has been extensively studied. Cytokine therapy (e.g., IL-6, TNF-β) was once a mainstay for mRCC but has declined in use due to low response rates and high side effects.^71^ Since 2015, immune checkpoint inhibitors (ICIs), mainly including two types: PD-1/PD-L1 (programmed death-1 receptor/programmed death-1 receptor-ligand) and CTLA4 (cytotoxic T lymphocyte-associated antigen-4), have revolutionized RCC treatment.^72^ Various combinations of targeted drugs and ICIs are recommended by RCC guidelines. Focal treatment such as stereotactic ablative radiotherapy for oligometastatic lesions is also essential for reducing tumor burden and prolonging survival.^71^^,^^73^ Despite these advances, therapy resistance remains inevitable. Later in the discussion below, we emphatically discuss the metabolic reprogramming in targeted drugs, ICIs, and radiotherapy resistance (Figure 3).

### Targeted drug resistance

Metabolic alterations represent a significant underlying mechanism of TKI resistance in RCC. TKI-resistant cells undergo significant bioenergetic adaptations to survive therapeutic challenge, which can be elucidated through metabolomic analyses comparing resistant cells with their parental counterparts.^74^

Specifically, glycolytic pathways are significantly augmented in TKI-resistant RCC cells. Metabolomics reveals that TKI-resistant RCC cells exhibit enhanced glycolysis, with increased glucose consumption and lactate production, and decreased excretion of TCA cycle-related organic acids.^74^^,^^75^ Disruptions in nicotinate and nicotinamide metabolism and reduced intracellular NAD+ are also observed in resistant RCC cells.^74^ NAD+ plays an important role in regulating the Warburg effect. Phosphoglycerate kinase 1 (PGK1), a critical glycolytic enzyme that catalyzes the reversible transfer of a phosphate group from 1,3-bisphosphoglycerate to ADP, producing 3-phosphoglycerate and ATP, is upregulated in tumor tissues and serum of patients with kidney renal clear cell carcinoma (KIRC) and correlates with poor prognosis. PGK1 promotes sorafenib resistance in KIRC by enhancing CXCR4-mediated ERK phosphorylation.^19^ PGK1 inhibitors, CHR-6494 and Z57346765, showed potent anti-KIRC effects *in vitro* and *in vivo*.^76^ PFKFB3 (6-phosphofructo-2-kinase/fructose-2,6-biphosphatase 3) served as a pivotal glycolysis regulator and mediates sunitinib resistance in patients with papillary RCC. Blocking PFKFB3 with the selective inhibitor PFK-015 significantly restrains papillary RCC cell neoplastic potential, and PFK-015 synergizes with sunitinib to suppress proliferation.^77^

Lipid metabolism is dysregulated in resistant cells, with accumulation of fatty acids, monoglycerides, and cholesteryl esters.^78^ Glycerophosphocholine and phosphatidylethanolamines, the major components of cell membranes, are dysregulated, supporting the greater proliferative ability of resistant cells. Lipid-rich RCC cells compensate for energy production and sustain growth independently of glycolysis, evading the cytotoxic effects of targeted drugs and fostering resistance. Mesoderm induction early response 2 (MIER2) promotes RCC malignancy and sunitinib resistance by influencing lipid metabolism. Disturbing the MIER2/HDAC1/P53/PGC1A pathway may benefit patients with sunitinib-resistant RCC.^79^ A deficiency in malonyl-CoA decarboxylase (MLYCD) facilitates fatty acid synthesis and lipid droplet accumulation, driving progression. Restoring MLYCD reduces tumor growth and reverses resistance to sunitinib *in vitro* and *in vivo*.^80^ Activating MLYCD-mediated fatty acid metabolism may be a promising therapeutic strategy for treating RCC. Other findings include lysophosphatidic acid reversing temsirolimus effects by increasing lipid droplets in a MAPK-dependent manner, and the SLC27A3/PINK1 pathway remodeling lipid metabolism to contribute to pazopanib resistance.^81^

Beyond glucose and lipid metabolism, reprogramming of amino acid metabolism also contributes significantly to TKI resistance. The up-regulation of several amino acids (glycine, aspartate, glutamate, isoleucine, alanine) was identified in resistant cells. Related pathways, including aminoacyl-tRNA biosynthesis and branched-chain amino acid degradation, also changed. Notably, increased demand for glutamine (also called glutamine addiction) and glutaminase up-regulation are distinct changes in RCC contributing to sunitinib and pazopanib resistance.^74^ Glutathione levels are significantly elevated in sunitinib-resistant RCC cells with enhanced antioxidant capacity.^82^ Taurine is a non-essential amino acid with anti-tumor properties.^83^ Sunitinib treatment induces serine synthesis in RCC cells via the activation of the GCN2-eIF2a-ATF4 pathway.^84^ Inhibiting the key enzymes in serine synthesis restrained the sensitivity of resistant RCC cells to sunitinib. MTHFD2, a mitochondrial enzyme in one-carbon metabolism, promotes sunitinib resistance by enhancing c-MYC O-GlcNAcylation, revealing a link between one-carbon metabolism and sunitinib resistance.^85^

### Immunotherapy resistance

RCC metabolic reprogramming creates a multifaceted immunosuppressive TME that drives immunotherapy resistance through nutrient competition, oncometabolite accumulation and metabolic crosstalk, impairing anti-tumor immunity.

Specifically, enhanced aerobic glycolysis in normoxia results in lactate accumulation in RCC cells, acidifying the TME. Lactate upregulates CTLA-4 on T cells, inhibits NFAT signaling, impairs T cell cytotoxicity and NK-cell function, and promotes M2 macrophage polarization.^86^ Furthermore, tumor cells overexpress GLUT1, depleting glucose and impairing glycolytic flux in CD8^+^ T cells, reducing IFN-γ production and cytotoxic function.^87^^,^^88^ Reduced ATP yield starves immune cells and creates an immunosuppressive niche. Beyond glycolysis, impaired mitochondrial metabolism contributes to resistance. In hereditary leiomyomatosis and renal cell cancer (HLRCC), FH loss leads to fumarate accumulation, inhibiting prolyl hydroxylase (PHD) and stabilizing HIF-1ɑ.^89^ SDH deficiency similarly drives pseudohypoxia. Impaired OXPHOS enhances glycolytic dependency. Accumulated oncometabolites (e.g., fumarate, succinate) cause oxidative stress and DNA damage, promoting mutagenesis.^90^^,^^91^^,^^92^ Another key mechanism involves the PPP. G6PD, the rate-limiting enzyme of the PPP, is associated with immunotherapy resistance. G6PD activation alleviated the efficacy of IFN-α agents. G6PD inhibition activated AMPK signaling, suppressing ACC1 enzyme and lipid synthesis. In addition, 6PGD inhibition reduced NADPH, NADH and SIRT-1 activity.^23^

Amino acid metabolism is also critically reprogrammed to drive immunosuppression. Glutamine deprivation in the TME can induce PD-L1 expression on tumor cells via the EGFR/ERK/c-Jun pathway.^93^ RCC cells increase glutamine uptake via ASCT2 transporters, limiting availability for T cell activation and promoting differentiation into exhausted PD-1+ subsets.^27^ IDO and TDO enzymes convert tryptophan to kynurenine, activating the aryl hydrocarbon receptor (AhR). This promotes regulatory T cell (Treg) differentiation and suppresses CD8^+^ T cell activity, inducing PD-1 expression and anergy.^94^ IFNα therapy failure for RCC may be due to its inability to counter the IDO1-mediated immunosuppressive environment. Compared to IFNα alone, the combination of IDO1 inhibitor methyl-thiohydantoin-DL-tryptophan (MTH-trp) and IFNα reduces tumor growth.^95^ In clinical specimens, TDO expression correlates with ICI response and survival in patients with mRCC and may predict primary immunotherapy resistance.^96^

Additional cellular players further reinforce this immunosuppressive milieu. Myeloid-derived suppressor cells (MDSCs) express arginase, depleting arginine and impairing T cell proliferation and memory responses.^97^^,^^98^ Finally, adenosine generated from ATP by CD39/CD73 enzymes on tumor cells suppresses dendritic cell maturation and T cell responses.^99^ Targeting fatty acid metabolism affects anti-PD-1/PD-L1 therapy efficacy. A twenty-gene fatty acid metabolism signature from TCGA stratifies patients into risk groups, with high-risk patients showing inferior responses to anti-PD-1/PD-L1 therapy.^100^

### Radiotherapy resistance

Metabolic reprogramming in RCC drives radiotherapy resistance through enhanced antioxidant defense, DNA repair, immunosuppressive TME remodeling and metabolic heterogeneity promoting cancer stem cells.

A primary mechanism is the creation of a metabolic barrier that directly protects tumor cells and suppresses anti-tumor immunity. Tumor-derived lactate accumulation acidifies the TME, impairing free radical formation during irradiation and reducing DNA damage efficacy.^101^^,^^102^ Lactate also abrogates radiation-induced immunogenic cell death by promoting M2 macrophage polarization and inhibiting CD8^+^ T cell cytotoxicity via CTLA-4 upregulation. Enhanced PPP flux increases NADPH production, boosting antioxidant capacity (e.g., glutathione) to neutralize radiation-induced ROS. RCC cells use glutamine to fuel reductive carboxylation, replenishing TCA intermediates for lipid biosynthesis under hypoxia. This pathway generates α-ketoglutarate, stabilizing HIF-1α and promoting DNA repair enzyme expression.^4^ Radioresistant RCC cells exhibit OXPHOS dependency and enhanced FAO. The immunosuppressive TME is further reinforced by specific metabolic pathways. IDO/TDO activation depletes tryptophan and produces kynurenine, activating AhR, inducing T cell exhaustion, and recruiting immunosuppressive MDSCs, dampening radiation-induced immune responses.^103^ Finally, profound metabolic heterogeneity within tumors creates physical and functional barriers that limit radiotherapy efficacy. Perivascular niches show high cholesterol efflux and glutamine metabolism, while hypoxic cores exhibit glycolytic dominance, creating heterogeneity that limits radiotherapy penetration and efficacy.

## Strategies to overcome therapy resistance in RCC

Metabolic reprogramming plays a central role in the progression and therapeutic resistance of RCC. Exploiting tumor-specific metabolic vulnerabilities has therefore emerged as a promising paradigm to circumvent resistance to conventional treatments. This section discusses four multifaceted strategies: (1) novel agents targeting core metabolic pathways; (2) rational combinations that co-inhibit complementary pathways to prevent compensatory adaptation; (3) metabolomics-guided precision therapy leveraging metabolic heterogeneity for patient stratification; and (4) nanoparticle-based delivery systems designed to enhance drug targeting and overcome systemic toxicity.

### Novel drugs targeting RCC cell metabolism

In recent years, several novel drugs have been investigated in preclinical *in vivo* and clinical studies (Table 1) to exploit metabolic vulnerabilities and improve therapeutic outcomes in RCC.^4^^,^^26^ Given the pivotal regulatory role of the VHL/HIF axis in the progression and therapeutic resistance of RCC, an increasing number of therapeutic agents targeting HIF-2α have been developed and translated into clinical practice. Belzutifan is the first-in-class HIF-2α inhibitor (FDA-approved), which suppresses its downstream transcriptional programs, normalizes glycolytic flux, and modulates PD-L1 expression.^115^ Owing to the central regulatory function of FASN in lipid metabolism, a next-generation FASN-targeting agent has demonstrated promising antitumor efficacy in a broad spectrum of solid malignancies.^116^ Regrettably, investigations in RCC remain confined to earlier-generation compounds, warranting further dedicated research. Additionally, as a polyether ionophore antibiotic, salinomycin has demonstrated potent anti-tumor efficacy against RCC. Mechanistically, salinomycin transcriptionally represses SREBP1 and thereby reverses sorafenib resistance by facilitating ferroptosis.^117^^,^^118^^,^^119^ Therapeutic strategies targeting amino acid metabolism have garnered substantial research interest, among which glutamine metabolism represents the most extensively investigated metabolic axis in RCC. Glutaminase-targeted inhibitors exhibit favorable pharmacokinetic profiles and promising preliminary antitumor efficacy (Table 1). In addition, the transglutaminase 2 inhibitor markedly abolishes RCC outgrowth in ACHN and CAKI-1 xenograft tumor models via p53 stabilization.^120^^,^^121^ Notably, as a key metabolic feature of ccRCC, glutamine addiction fuels tumor progression and remodels the tumor immune microenvironment to trigger immune evasion.^122^^,^^123^ Research targeting the metabolism of other amino acids, including tryptophan, arginine and serine, is also being progressively advanced.^124^^,^^125^^,^^126^

**Table 1:** Current drugs targeting metabolism pathways in RCC

Molecular target	Pharmaceutical	Experience classification	Results
HIF-2	PT2399	in preclinical model	inhibits tumor growth.^104^
	PT2385	first-in-class HIF-2 inhibitor, Phase I clinical trial	PT2385 has a favorable safety profile and is active in patients with heavily pretreated ccRCC.^105^
	PT2977(Belzutifan)	first-in-class HIF-2 inhibitor, FDA-approved	belzutifan showed a significant benefit over everolimus with respect to progression-free survival and objective response in participants with advanced ccRCC who had previously received immune checkpoint and antiangiogenic therapies.^106^
FASN	C75	in preclinical model	inhibit tumor growth.^107^
PPARα	GW6471	in preclinical model	inhibit tumor growth.^108^
Glutaminase	Telaglenastat (CB-839)	clinical trial	TelaE (telaglenastat plus everolimus) and TelaC (telaglenastat plus cabozantinib) showed encouraging clinical activity and tolerability in heavily patients with pretreated mRCC.^109^
	BPTES	in preclinical model	BPTES synergistically inhibits VHL-deficient renal cell carcinoma when combined with PARP inhibitors.^110^
ASCT2(SLC1A5)	V9302	in preclinical model	inhibits tumor growth.^111^
IDO1	Epacadostat	clinical trial	epacadostat in combination with pembrolizumab was generally well tolerated and had encouraging antitumor activity.^112^
	Navoximod	clinical trial	the combination of navoximod and atezolizumab demonstrated acceptable safety, tolerability, and pharmacokinetics.^113^
NADP/NADPH	KPT-9274 (PAK4/NAMPT inhibitor)	in preclinical model	inhibits tumor growth.^114^

Overall, novel drugs targeting metabolic reprogramming are highly anticipated. However, most candidates entering clinical trials fail to yield satisfactory therapeutic effects, primarily due to rapid compensatory pathway activation and a lack of biomarker-guided patient stratification.

### Combination of different therapies

The combination of different therapies co-inhibiting complementary pathways creates synergy and prevents compensatory adaptation. Following the superior efficacy of belzutifan versus everolimus as second-line therapy for advanced ccRCC, multiple combination regimens based on belzutifan have been successively investigated. Results from the LITESPARK-003 trial (cohort 1) demonstrated that the combination of belzutifan and cabozantinib yields promising antitumor activity with manageable safety profiles in treatment-naive patients with advanced ccRCC.^127^ Notably, the recently published LITESPARK-022 trial provided novel clinical evidence: the combination of pembrolizumab and belzutifan further reduces recurrence risk compared with single-agent pembrolizumab, and this regimen has been approved by the FDA as the new standard adjuvant therapy for ccRCC.^128^ The limited clinical benefit of immunotherapy in certain contexts may be attributed to metabolic dysregulation-induced immune evasion. A preclinical study demonstrated that αKG supplementation combined with PD-1 blockade improved therapeutic efficacy and prolonged survival in murine models compared with monotherapy. This synergistic effect was mediated by elevated αKG levels, which promoted major histocompatibility complex-I (MHC-I) antigen processing and presentation, as well as β2-microglobulin (B2M) expression.^13^

Several studies have evaluated the synergy of telaglenastat (a selective glutaminase inhibitor) and other drugs, including everolimus and cabozantinib, for patients with treatment-refractory RCC. Compared to everolimus plus placebo, everolimus plus telaglenastat improved PFS in patients with mRCC previously treated with TKIs and ICIs (3.8mon vs. 1.9mon).^120^^,^^129^ Intriguingly, a separate study demonstrated that pharmacological suppression of glutamate-ammonia ligase (GLUL) via L-methionine sulfoximine not only attenuates lipid deposition and tumor growth in RCC but also potentiates the anticancer activity of everolimus.^130^ This reminds us that, given the complexity of metabolic reprogramming, more attention should be paid to the dynamic alterations of metabolic networks and the compensatory crosstalk between upstream and downstream metabolic enzymes, which is essential for guiding the development of precise and efficient metabolically targeted therapeutic strategies for RCC. Disordered glutamine metabolism and lipid accumulation jointly trigger mitochondrial dysfunction, a typical metabolic hallmark of RCC. As a core mitochondrial deacetylase, SIRT3 maintains mitochondrial homeostasis and potently inhibits the Warburg effect.^131^ One preclinical study found that overexpression of SIRT3 combined with resveratrol produces synergistic cytotoxic effects in ccRCC,^20^ reminding us that targeting mitochondrial metabolism to reverse glycolytic disorders may serve as a viable therapeutic strategy.

### Metabolomic-guided precision therapy

**Figure 4:**
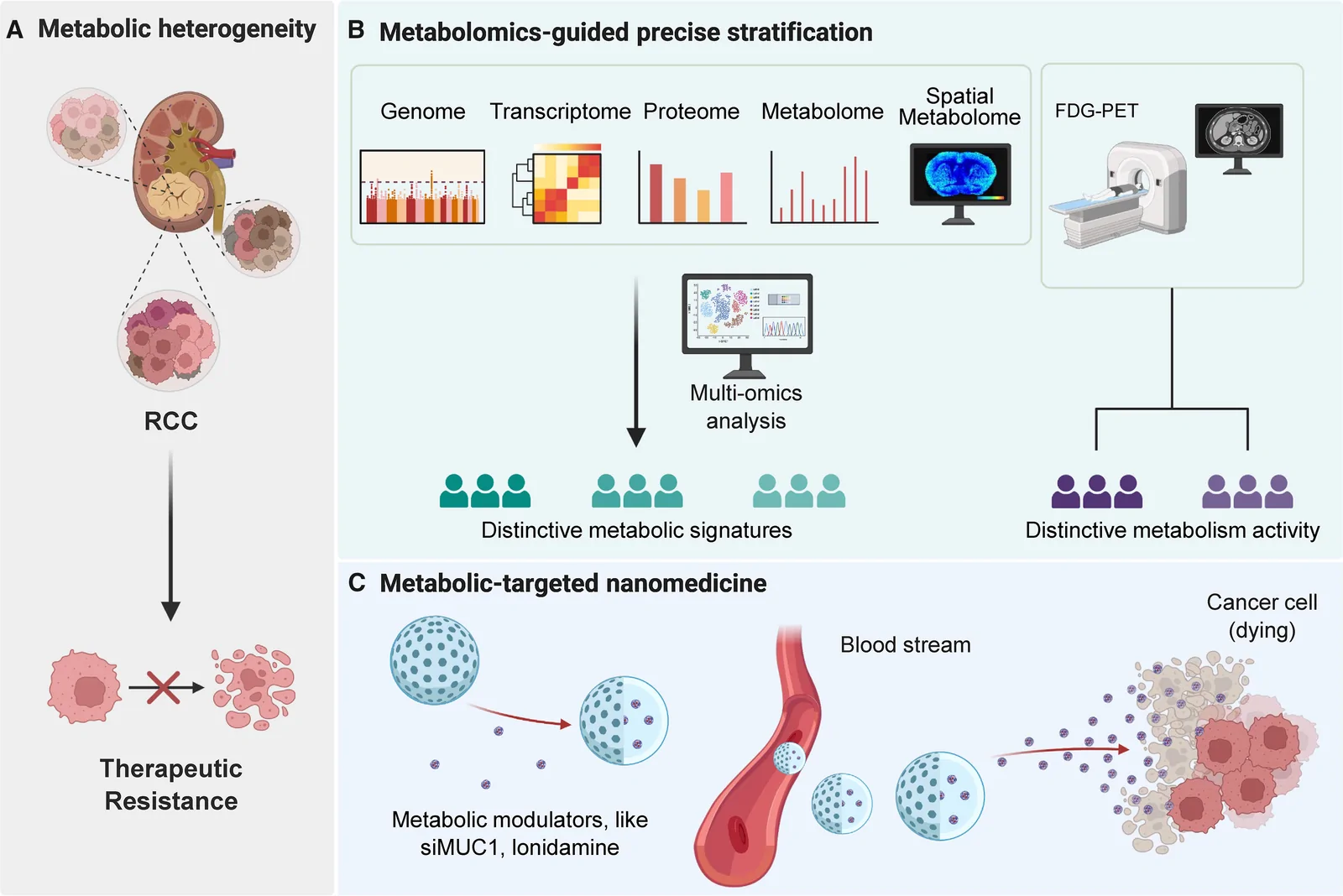
Schematic diagram illustrating metabolomics-guided precise intervention and metabolically targeted nanomedicine to reverse therapeutic resistance in RCC (A) Metabolic heterogeneity drives therapeutic resistance in RCC. (B) Metabolomics-guided precise patient stratification. (C) Metabolic-targeted nanomedicine intervention strategy.

Metabolic heterogeneity exists between different individuals even in the same tumor entity, which is one of the main mechanisms for RCC resistance (Figure 4A). Metabolomics enables comprehensive profiling of small-molecule metabolites, offering a robust approach to deciphering tumor-specific metabolic dysregulation. Metabolome-based stratification further classifies RCC into distinct molecular subtypes to facilitate precision treatment.^4^ Biomarker-driven patient stratification and rational combinations exploit metabolic vulnerabilities while minimizing toxicity.

Driven by technological and methodological advances in mass spectrometry imaging, spatial metabolomics involves the profiling of all metabolites so that spatial information is captured within the sample and offers an opportunity to demonstrate the drug-resistant RCC tumor profile with metabolic heterogeneity. With multi-omics detection and single-cell and spatial trajectory analysis, four clear cell RCC subtypes, including de-differentiated clear cell RCC (DCCD), were identified. DCCD cancer cells are characterized by fewer lipid droplets, reduced metabolic activity, enhanced nutrient uptake capability, and a high proliferation rate.^132^ These patients require more aggressive intervention due to poor prognosis, even at early disease stages.

Analyzing RCC metabolic fingerprints allows real-time identification of resistance mechanisms, prediction of treatment outcomes, and rational selection of combination therapies to overcome resistance, paving the way for precision medicine.^133^ FDG-PET can detect the upregulation of GLUT1 in glycolysis-active tumors, enabling precise patient screening and stratified treatment. Given the extensive intratumoral metabolic zonation in RCC, personalized combinatorial therapeutic strategies are urgently required to target spatially heterogeneous tumor phenotypes. Spatial metabolomics maps the metabolic gradients (e.g., pseudohypoxic vs. vascular niches) to identify regional vulnerabilities. Collectively, the integration of spatial metabolomics with biomarker-based patient stratification holds great potential to reshape the current landscape of RCC clinical management and improve therapeutic efficacy (Figure 4B).

### Nanoparticles co-loading with anti-tumor metabolism drugs

Conventional anti-tumor drugs often face challenges such as systemic toxicity and drug resistance. Nanoparticle (NP) drug delivery systems offer a promising solution by enabling targeted delivery of therapeutic agents directly to the tumor site, enhancing efficacy while minimizing side effects.^134^ They are specifically engineered to exploit the unique metabolic vulnerabilities of RCC (Figure 4C). A nanomicelle delivery system named SPPI comprising polyethyleneimine (PEI), ibuprofen, and sialic acid can successfully deliver siRNA to cancer cells by overcoming systemic and cellular barriers. SPPI/siMUC1 nanomicelles demonstrated outstanding growth inhibition of sunitinib-resistant 786-O cell xenografts and remarkable inhibitory effects on tumor metastasis via suppression of MUC1 expression and a decrease in cholesterol synthesis. It subsequently reversed resistance to sunitinib by modulating P-GP, ABCG2 and MRP1 activities.^135^ Another system, EMφ-siMTHFD2-MnO2@Suni, was designed to simultaneously target CD276 and CD133 and remodel folate-nucleotide and GABA metabolism, thereby overcoming sunitinib resistance in RCCs.^136^ Lonidamine (LND) is a thermosensitive anticancer drug, inhibiting hexokinase activity. LND and photothermal material polydopamine (PDA) were loaded in stellate mesoporous silica nanoparticles (MSNs) with a large surface area and cloaked with RCC membranes (called MLP@M). MLP@M exhibited excellent antiproliferative and tumor-suppressing abilities when triggered by an 808 nm laser.^137^

## Conclusion

Metabolic reprogramming is a hallmark of RCC progression and therapy resistance. Targeting metabolic vulnerabilities—including glycolysis, lipid metabolism, glutamine dependency, and serine biosynthesis—offers novel opportunities to enhance therapeutic efficacy. Although challenges such as intratumoral heterogeneity and systemic toxicity remain, emerging strategies including rational combination regimens, metabolomics-guided precision medicine, and nanoparticle-based delivery systems provide new avenues for clinical translation. Ultimately, the identification of tumor-specific metabolic “Achilles heels” may enable real-time adaptive treatment strategies, bringing renewed hope for patients with advanced and treatment-refractory RCC.

## Acknowledgments

This study was supported by the Clinical Medicine Research Special Project (2024LYA05).

## Author contributions

Conceptualization, J.Z.S., L.H.W., and X.X.G.; writing – original draft preparation, J.Z.S., H.W., X.X.G., and R.Y.W.; visualization, H.W., S.Y.W., and J.T.H.; writing—review and editing, J.Z.S. and L.H.W.

## Declaration of interests

The authors declare no conflict of interest.
